# Acute distal biceps tendon rupture: retrospective analysis of two different approaches and fixation techniques

**DOI:** 10.1007/s00590-021-03132-8

**Published:** 2021-10-01

**Authors:** Marco Di Stefano, Lorenzo Sensi, Leonardo di Bella, Raffaele Tucci, Efisio Bazzucchi, Luigi Zanna

**Affiliations:** Department of Shoulder and Elbow, University of Florence, A.O.U. Careggi CTO - Largo Palagi 1, 50139 Florence, Italy

**Keywords:** Distal biceps tendon rupture, Single-incision technique, Double-incision technique, Functional and clinical outcome, Complications, Range of motion

## Abstract

**Purpose:**

The aim of our study is to compare the modified double incision (DI) with bone tunnel reinsertion with the single-incision (SI) double tension slide technique in terms of clinical and functional outcomes and complication rates.

**Methods:**

A retrospective comparative analysis was performed on 65 patients treated for total distal biceps tendon rupture. The surgical technique adopted for each patient was based on the preference of two experienced elbow surgeons. The DASH and MAYO questionnaires, functional outcome and ROM were recorded in all subjects.

**Results:**

Of 65 patients, we collected data of a cohort of 54 distal biceps tendon ruptures that satisfied inclusion criteria. Twenty-five were treated by modified DI and 29 SI techniques. The recovery of the complete ROM in terms of flexion/extension and prono-supination occurred in the 79.6% of the patients, without statistical significant difference between the adopted technique. We reported a complication rate of 12% and 20.7% for DI and SI techniques, respectively, without statistical correlation (*P* = *0.84*). The average DASH score was similar for DI and SI techniques without significant differences (*P* = 0,848). The Mayo score results were excellent in the majority of the patients. No significant difference in MAYO results was reported comparing the surgical techniques (*P* = *1*).

**Conclusion:**

Both techniques provide a reliable and strong repair with an optimal recovery of ROM returning to preinjury activity with substantially overlapping timelines.

## Introduction

Distal biceps tendon ruptures are an uncommon injury, representing 3% of all tendon lesions [1, 2], with an incidence of 2.5 per 100,000 persons per year [3]. Two-third of distal biceps tendon ruptures occur in active middle-aged male [3]. The decreased range of motion (ROM) of strength and chronic pain are the most common functional deficits resulting of conservative treatment [4]. Primary repair with reinsertion of the biceps tendon into the radial tuberosity showed good restoration of strength and pain resolution [5]. Nonoperative treatment is an option in elderly or sedentary lifestyle people or in case of surgical contraindication [6]. Several surgical approaches and fixation techniques are reported in the literature [7]. Early repair can be performed through a single-anterior-incision technique or a two-incision technique. Numerous fixation techniques, such as suture anchors, bone tunnels, interference screws or cortical buttons, have been developed. Nonetheless, in the current literature, there is no clear consensus regarding the optimal surgical techniques [8]. The choice of the surgical technique is currently driven by surgeon preference. We hypothesized that the DI and SI techniques would lead to functionally equivalent results. The aim of our study is to compare the modified double incision (DI) with bone tunnel reinsertion with the single-incision (SI) double tension slide technique in terms of clinical and functional outcomes and complication rates.

## Materials and methods

A retrospective comparative analysis was performed on 65 patients treated for total distal biceps tendon rupture from July 2016 to January 2020 in our Shoulder and Elbow Surgery Unit. Inclusion criteria were patients with complete distal biceps tendon rupture surgically treated within 4 weeks from the time of injury and a postoperative follow-up (FU) more than 12 months. Additionally, the tendon repair had to be performed using either the SI or the DI technique. The partial ruptures, chronic lesions, patients treated more than 30 days after the initial trauma and those with less than 1 year of FU were excluded (*x* = 11). The primary endpoints of our study are the clinical outcomes defined as MAYO and DASH scores, the recovery of flexion–extension and prono-supination ROM. The secondary endpoint is the complication rate. All patients admitted to our Emergency Department underwent a detailed clinical examination and radiological assessment. The diagnosis of acute total rupture was suspected after the accurate orthopedic physical assessment, with the squeeze and hook tests. The complete rupture was confirmed by ultrasound (US). Considering the traumatic mechanism, in 36 patients, radiographs were performed to exclude elbow fractures or bone avulsions. Magnetic resonance imaging was performed in 10 cases, to confirm the diagnosis or quantify the lesion. The surgical technique was adopted according to the preference of two orthopedic surgeons (E.B. and R.T.) who have the expertise and experience for high-quality surgical results in both SI and DI procedures.

### Surgical technique

**Fig.1:**
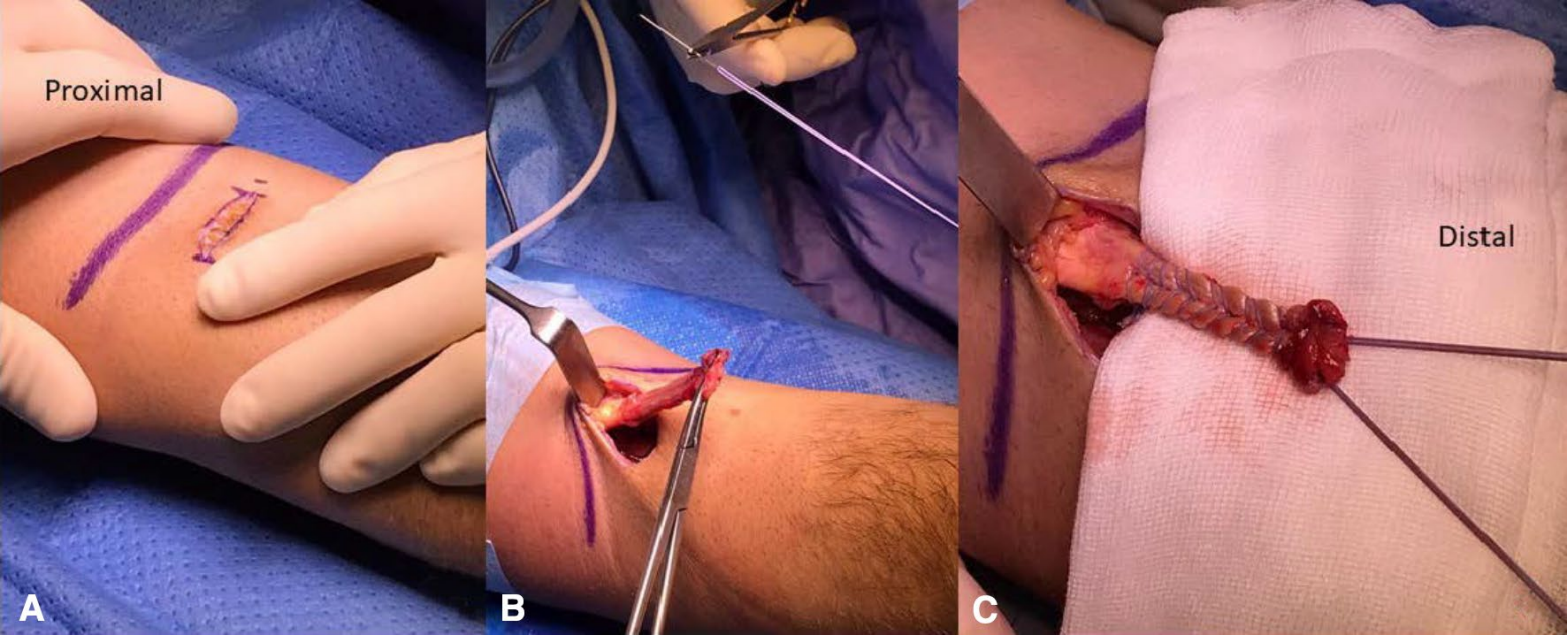
**a** Anterior transverse incision distal to the flexion crease of the antecubital fossa. **b** Retracted distal biceps tendon after the identification and mobilization until myotendinous junction. In the upper part, the straight needle used for harnessing the whole tendon. **c** Distal 3.5 cm harnessed with 2 suture wires using Bunnell techniques. 2 central and 2 lateral strands exit at the distal aspect of the tendon

All the patients were placed in supine position, under regional plexus anesthesia. A tourniquet was applied on the proximal arm. We identified the retracted distal biceps tendon and the radial tuberosity using US before starting surgery. An anterior limited transverse incision was routinely performed 2 cm distal to the flexion crease of the antecubital fossa for both techniques (Fig. 1a). After blunt dissection of superficial tissue with particular care to the lateral antebrachial cutaneous nerve (LABCN), the retracted distal end of the biceps tendon was identified. The tendon was mobilized, and the myotendinous junction was dissected and debrided (Fig. 1b). The 35-mm distal part of the whole tendon was harnessed with No. 2.0 Ethibond Excel® (Ethicon®, New Jersey, USA) using a Bunnell suture. The first suture was made with a straight needle in the central portion of the tendon. We began 35 mm proximally to the end of the tendon, running distally and exiting with 2 central strands. This procedure was repeated using a second suture, ending laterally at the distal aspect of the tendon (Fig. 1c). Finally, we had 2 central and 2 lateral sutures. The radial tuberosity and biceps tendon insertion were identified after the laterally and medially retraction of brachioradialis and pronator teres, respectively, through the muscle interval between the brachioradialis and the flexor carpi radialis.

#### Double-incision technique

**Fig. 2:**
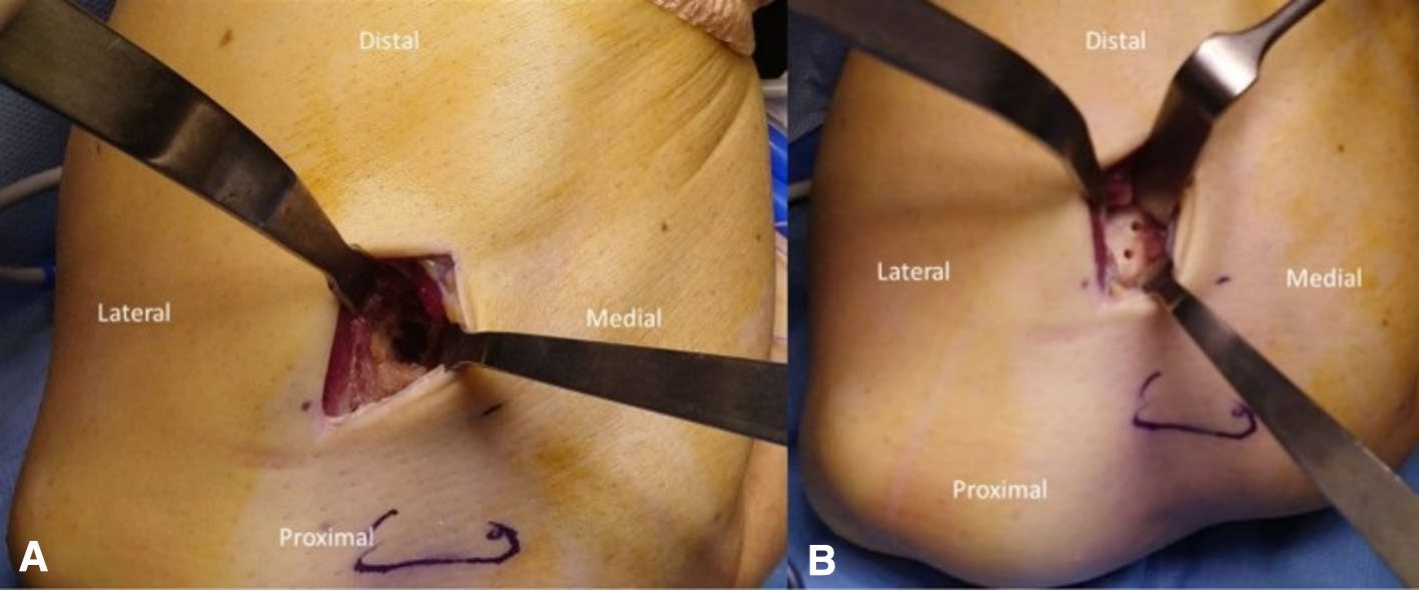
**a–b** DI Technique: (**a**) The bony trough in the ulnar aspect of the bicipital tuberosity, to allow the tendon to be docked into the radius (1.5 cm wide and 1 cm deep); (**b**) two 2.0-mm transosseous holes drilled in the dorsal cortical margin of the tuberosity 10 mm apart and 2 mm away from the cortical edge

In the DI technique, a pair of curved forceps were placed in the interosseous radioulnar space. During this maneuver, the forearm had to be held in complete pronation in order to prevent the damage to the posterior interosseous nerve (PIN) and ulnar cortex. The second incision was made after identification of the forceps’ tip under the skin on the dorsal aspect of the proximal forearm. The radial tuberosity was exposed dorsally through lateral muscle-splitting approach between extensor ulnaris carpi and extensor digitorum communis. The radial tuberosity was debrided, and a burr was used to create a trough in the ulnar aspect of the bicipital tuberosity. The trough was 1.5 cm wide and 1 cm depth to allow the tendon to be docked into the radius (Fig. 2a). Two 2.0-mm transosseous drill holes were drilled in the dorsal cortical margin of the tuberosity 10 mm apart and 2 mm away from the cortical edge (Fig. 2b). The tendon was passed through the anterior approach to the second incision. The 4 suture limbs were passed through the two 2.0-mm holes, two in the proximal and two in the distal one. Finally, the biceps tendon was pulled into the bicipital tuberosity and the sutures were tensioned, with the elbow flexed at 90° and the forearm in mild supination in order to prevent excessive tension. The 2 central and the 2 lateral sutures were tied over the bone bridge. A copious irrigation was performed to remove all bone debris to reduce the risk of heterotopic ossification (HO).

#### Single-incision technique

**Fig. 3:**
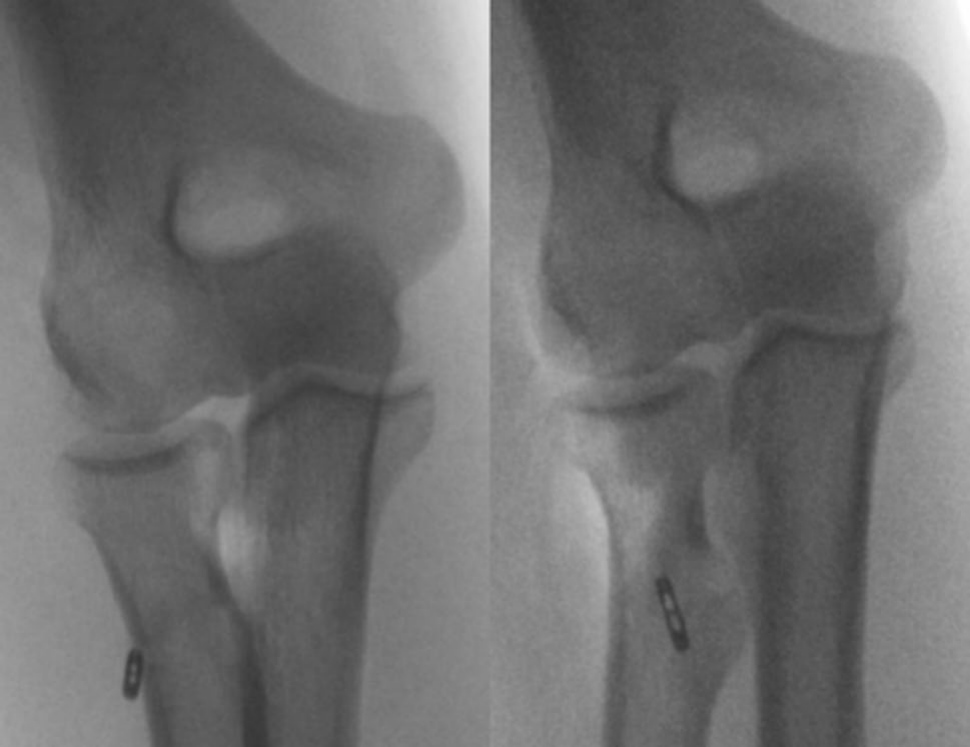
SI technique. Biceps button “flipped” against the radial posterior cortex, under intraoperative fluoroscopic control. Antero-posterior views in pronation and supination position

In SI technique, a 3.2-mm guide pin was drilled through the central radial tuberosity cortex from anterior to posterior, under fluoroscopy control. The anterior cortex and intramedullary were reamed with an 8.0-mm cannulated reamer to allow the tendon to be pulled onto the tuberosity. Meticulous irrigation was routinely performed to remove bone dust and fragments. The 2 central strands of the sutures are then threaded through the cortical button (BicepsButton, Arthrex) in opposite directions. In the same way, the 2 lateral strands were encircled, however, in the opposite direction of the first two. In such a way, both sutures were facing toward the distal biceps tendon on the anterior radial cortex. The button was then released from the holder and “flipped” against the radial posterior cortex, under fluoroscopic control (Fig. 3). Therefore, the 2 limbs of suture passed through the button were toggled to dock the tendon into the bone socket. Once the tendon was fully seated in the socket, the 2 limbs of the same suture were tied together for both the medial and lateral sutures. The sutures were passed through the tendon with a free needle, proximally to the new insertion, and tied to reinforce the construct.

### Rehabilitation

All patients underwent the same rehabilitation protocol. Indomethacin was not administered. Articulated elbow brace was placed in neutral prono-supination position for one month. Since the first week, passive flexion/extension exercises with restricted ROM (0°–90°) were encouraged in order to avoid pain and promote soft tissue healing. The ROM was progressively increased with the purpose of achieving the complete passive flexion/extension within the first 30 days. Assisted prono-supination movements were exclusively allowed with the flexed elbow without excessive tension to the tendon. Full active ROM was allowed after 4 weeks, and the complete flexion/extension and prono-supination were recovered after 6 weeks. After 8 weeks of non-weight bearing, patients were advanced to a 2-kg lifting restriction. Three months after surgery, they began progressive resistance exercises as tolerated.

### Postoperative evaluation

Clinical evaluation, including physical examination and functional outcomes’ assessment, was performed at 1, 3 and 12 months postoperatively. A postoperative antero-posterior and lateral radiograph of the elbow was routinely performed 1 months after surgery. At the last FU clinical evaluation, DASH and MAYO questionnaires were registered and flexion/extension and prono-supination ROM was assessed by standard goniometer by a surgeon of the Shoulder and Elbow Surgery Unit. All surgical complications, such as nerve damage, infection, soft tissue damage, heterotopic ossification, rerupture of the distal biceps tendon, and radioulnar synostosis, were recorded. Statistical analysis was performed using SPSS statistics software version 25.0 for MACINTOSH (IBM, Armonk, NY). The normal distribution was tested with the Kolmogorov–Smirnov’s (KS) test. Descriptive statistics (mean, standard deviation, etc.) were used to describe the patients’ variables and clinical outcomes. Categorical variables, such as Mayo score, complications and recovery of ROM, were assessed using the Chi-square test or Fisher exact test for statistical significance. *T* student test was used to evaluate the impact of the surgical techniques on Dash Score. *P*-values < 0.05 were considered statistically significant.

## Results

**Fig. 4:**
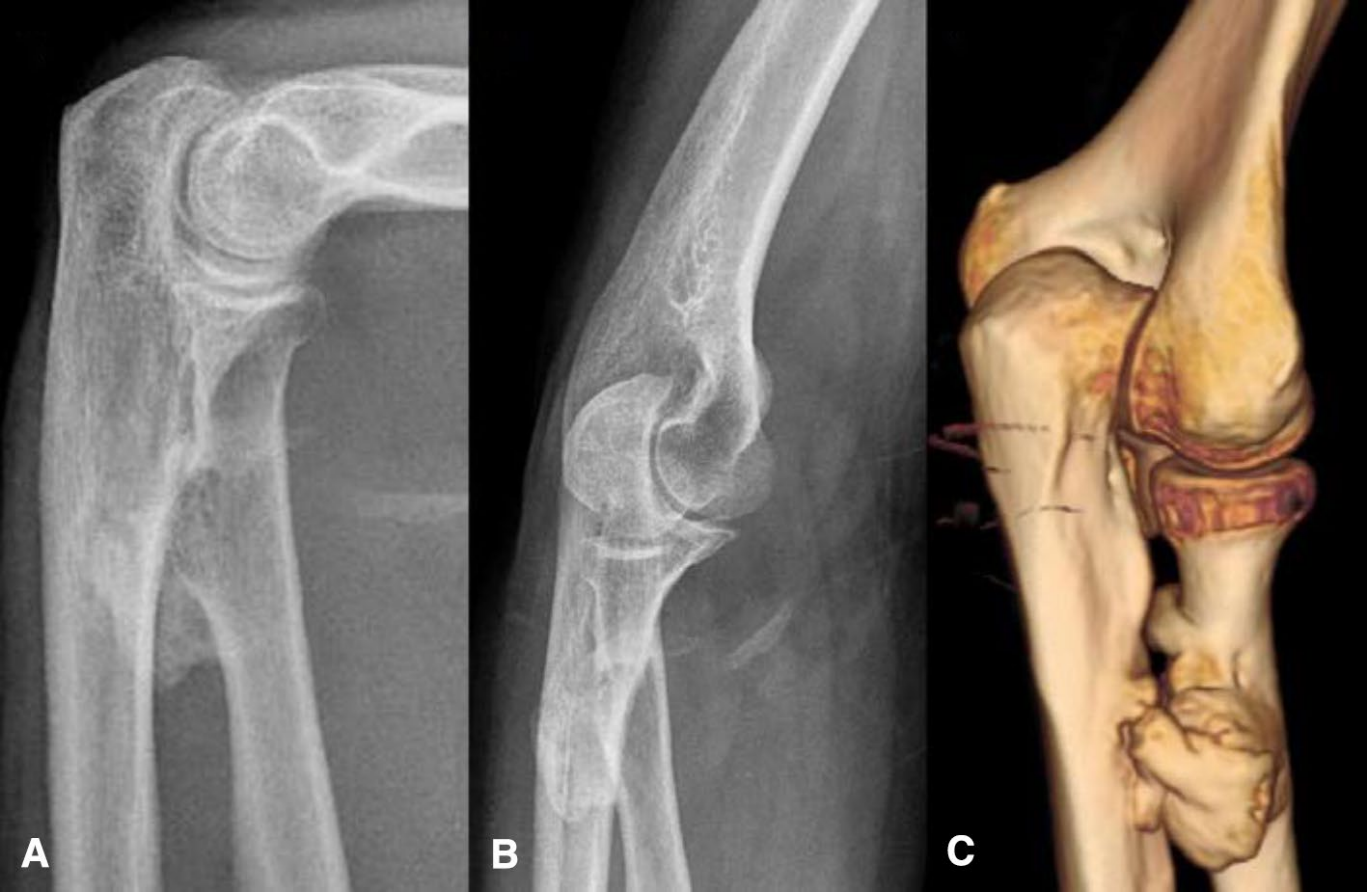
Proximal radioulnar synostosis: **a–b** antero-posterior and lateral X-ray views; **c** 3D TC reconstruction

Of 65 patients treated for total distal biceps tendon rupture, 51 were enrolled with a total of 54 distal biceps tendon ruptures (Table 1). All patients were male. The mean age was 48.85 ± 6.62 years showing a normal distribution on KS test analysis (*P* = 0.59). Right-side ruptures were observed in 20 patients (39.2%), left side in 28 (54.8%) and 3 had bilateral elbow involvement (6%). Dominant arm was affected in 25 cases (46.3%) and the non-dominant arm in 29 (53.7%). The FU was 26.1 ± 11.85 months, and the mean hospital stay was 1 day. The interval between the injury and surgery was 12.6 ± 6.9 days. Twenty-five patients underwent DI technique (46.3%) and 29 SI technique (53.7%). The normal flexion/extension and prono-supination ROM was 0°–145° and 85°–90°, respectively [9–11]. The recovery of the complete ROM in terms of both flexion/extension and prono-supination occurred in the 79.6% of the patients, as reported in Table 1. The recovery of complete flexion, extension, pronation and supination occurred in 94.4%, 96.3%, 88.9% and 90.7% of patients, respectively. No significant differences between the surgical techniques were reported regarding the recovery of the complete flexion–extension (*P* = 0.449) and prono-supination ROM (*P* = 0.137) (Table 2). No cases of tendon rerupture were observed during FU. Twelve percent of patients treated by DI technique and 20.7% treated by SI technique developed complications (Table 3). The case of proximal radioulnar synostosis with severe limitation of supination (Fig. 4) required a second surgical procedure in order to obtain a complete resolution of the symptoms. The patient that developed the wound infection was surgically treated with meticulous irrigation, debridement and antibiotic therapy. Comparing the two surgical techniques, we did not register any statistical difference concerning the risk to develop postoperative complications (*P* = 0.84). At final FU, the average DASH score of patients who underwent DI technique was 3.1 (0–26.9) and 2.9 (0–29.3) for SI technique. We did not find a statistically significant difference (*P* = 0.848) between the DASH results of two techniques. The MAYO score showed excellent results in the 80% of patients treated by DI technique and in the 79.3% of patients treated by SI technique. No significant difference in MAYO results was reported comparing the two surgical techniques (*P* = 1) (Table 4).

**Table 1:** Patients’ features include age, gender, side of elbow, follow-up, surgical technique, postoperatively ROM and time to surgery

N°	Age (y)	Gender	Injured side	Follow-up (Months)	Surgical technique	Postop. Pron-Sup ROM	Postop. Ext-Flex ROM	Time to surgery (days)
1	48	M	Right	20	Single Incision	85°–80°	0°–145°	22
2	62	M	Right	18	Single Incision	85°–90°	0°–145°	17
3	43	M	Right	43	Single Incision	85°–90°	0°–145°	9
4	44	M	Right	22	Single Incision	85°–90°	0°–132°	10
5	47	M	Left	12	Single Incision	50°–70°	0°–145°	6
6	40	M	Right	45	Single Incision	78°–90°	0°–145°	8
7	49	M	Right	42	Single Incision	85°–90°	0°–145°	9
8	57	M	Right	25	Single Incision	74–90°	0°–145°	22
9	48	M	Right	46	Single Incision	85°–90°	0°–145°	4
10	49	M	Left	32	Single Incision	85°–90°	0°–145°	29
11	53	M	Right	23	Single Incision	85°–79°	5°–145°	13
12	52	M	Right	21	Single Incision	85°–90°	10°–130°	18
13	49	M	Right	23	Single Incision	85°–90°	0°–145°	13
14	57	M	Left	21	Single Incision	85°–90°	0°–145°	10
15	48	M	Left	39	Single Incision	80°–90°	0°–145°	14
16	44	M	Left	44	Single Incision	85°–90°	0°–130°	7
17	54	M	Left	23	Single Incision	85°–90°	0°–145°	28
18	47	M	Left	28	Single Incision	85°–90°	0°–145°	14
19	27	M	Left	46	Single Incision	85°–90°	0°–145°	5
20	44	M	Left	33	Single Incision	85°–90°	0°–145°	10
21	47	M	Left	27	Single Incision	85°–90°	0°–145°	3
22	39	M	Left	22	Single Incision	85°–90°	0°–145°	18
23	55	M	Left	36	Single Incision	85°–90°	0°–145°	4
24	48	M	Right	17	Single Incision	85°–78°	0°–145°	14
25	49	M	Left	46	Single Incision	85°–90°	0°–145°	11
26	42	M	Left	33	Single Incision	85°–90°	0°–145°	18
27	49	M	Left	47	Single Incision	85°–90°	0°–145°	15
28	54	M	Right	34	Single Incision	85°–90°	0°–145°	21
29	44	M	Left	27	Single Incision	85°–90°	0°–145°	15
30	50	M	Left	43	Double Incision	85°–90°	0°–145°	5
31	56	M	Left	34	Double Incision	85°–90°	0°–145°	10
32	49	M	Left	46	Double Incision	85°–90°	0°–145°	4
33	55	M	Right	42	Double Incision	30°–0°	0°–145°	12
34	60	M	Right	26	Double Incision	85°–90°	0°–145°	11
35	58	M	Right	15	Double Incision	76°–80°	0°–145°	8
36	48	M	Right	12	Double Incision	85°–90°	0°–145°	1
37	46	M	Left	19	Double Incision	85°–90°	0°–145°	7
38	51	M	Right	12	Double Incision	85°–90°	0°–145°	3
39	43	M	Right	16	Double Incision	85°–90°	0°–145°	27
40	57	M	Right	19	Double Incision	85°–90°	0°–145°	6
41	41	M	Left	15	Double Incision	85°–90°	0°–145°	11
42	38	M	Right	13	Double Incision	85°–90°	0°–145°	7
43	48	M	Left	14	Double Incision	85°–90°	0°–145°	23
44	41	M	Left	12	Double Incision	85°–90°	0°–145°	3
45	47	M	Left	12	Double Incision	85°–90°	0°–145°	14
46	60	M	Left	13	Double Incision	85°–90°	0°–145°	17
47	59	M	Left	12	Double Incision	85°–90°	0°–145°	10
48	50	M	Left	15	Double Incision	85°–90°	0°–145°	10
49	44	M	Right	14	Double Incision	85°–90°	0°–145°	18
50	42	M	Left	22	Double Incision	85°–90°	0°–145°	9
51	53	M	Right	16	Double Incision	85°–90°	0°–145°	23
52	44	M	Left	14	Double Incision	85°–90°	0°–145°	13
53	52	M	Left	19	Double Incision	85°–90°	0°–145°	19
54	56	M	Left	37	Double Incision	85°–90°	0°–145°	21

ROM values are expressed in degrees.

**Table 2:** Final median and range of elbow and forearm ROM at final FU

	Single incision (SI) (*n* = 29)	Double incision (DI) (*n* = 25)	*P* value
	Median (range)	Median (range)	
Flexion	145° (130°–145°)	145° (145°–145°)	**0.449**
Extension	0° (0°–10°)	0° (0°–0°)	
Supination	90° (70°–90°)	90° (0°–90°) *	**0.137**
Pronation	85° (50°–85°)	85° (30°–85°)	

*0° of supination was registered in the patient that had developed the radioulnar synostosis

*P* value of the correlation between the surgical technique and complete recovery of ROM

**Table 3:** Complications rate reported in our populations according to the two surgical techniques

Complications	Double-incision technique	Single-incision technique
Superficial infections	1	0
Heterotopic ossification	1	3
Transient LACBN Palsy	0	3
R-U synostosis	1	0
	(3/25) **12%**	(6/29) **20.7%**

**Table 4:** Clinical outcomes defined as MAYO and DASH score according to the surgical technique performed. P value of the correlation between the surgical technique and both MAYO and DASH scores

Scores	Double incision technique		Single incision technique		*P* value
*MAYO SCORE*	96.8 (75-100)		96.1 (70-100)		**1**
		>90 20 Excellent		>90 23 Excellent	
		75-89 5 Good		75-89 5 Good	
				60-74 1 Fair	
		60-74 0 Fair		<60 0 Poor	
		<60 0 Poor			
*DASH SCORE*	3.1 (0-26.9)		2.9 (0- 29.3)		**0.848**

## Discussion

The surgical treatment of distal biceps tendon rupture provides superior results than conservative treatment in terms of functional outcome and strength recovery [4, 12]. There is still no consensus on the best surgical treatment for distal biceps tendon lesions [8, 13, 14]. We retrospectively compare the clinical outcomes and complication rate of patients treated with the SI and DI surgical techniques. The DI procedure, described by Boyd and Anderson [12] and modified by Morrey [4], provided a stable and strong fixation allowing a rapid rehabilitation. On the other hand, the SI double tension slide technique as reported by Sochacki et al. [15] preserved the advantages of the tension slide technique without the risk of bone tunnel fracture due to the positioning of the interference screw [15]. The tension slide procedure with a suspensory cortical button had a high resistance when compared with suture anchors, interference screw and transosseous techniques [16]. Sethi et al. reported that the use of interference screws was associated with an increased risk of fracture through the bone tunnels due to screw mobilization [17]. With this regard, we decided to avoid the positioning of the screw with the consequent reduction of the risk of fracture, and we performed a second suture. The addition of a second suture would likely increase the strength of the repair, but this had not been previously both investigated and demonstrated. Our monocentric experience may have a remarkable clinical relevance. It demonstrates successful use of either technique with a relatively low complication rate and similar satisfying clinical and functional outcomes. Both samples have optimal clinical results according to DASH and MAYO scores with no statistically significant differences. In the last decades, many authors have focused their efforts on the comparison between outcomes of the SI and DI techniques. Data reported comparable clinical and functional findings as well as similar surgical results, ability to recover preinjury level of functioning, and recovery of daily living activities [14, 18–20]. The DASH scores in our cohorts are slightly superior than those presented in other studies [19]. Castioni et al. [13] showed an average postoperative DASH score of 6.5 and 6.7 for SI and DI, respectively. The majority of our patients of both groups report a complete recovery of ROM. Between the two surgical procedures, no significant differences are found regarding the flexion–extension and prono-supination ROM. Additionally, Grewal et al. [19] registered comparable results with no significant differences in elbow ROM between the two analyzed populations. Conversely, Shields et al. [18] reported a recovery of flexion significantly greater in the SI than DI group and no significant differences regarding extension, supination and pronation. Moreover, Castioni et al. registered a greater recovery of both flexion and pronation in the SI technique compared to DI technique [13]. We register 16.7% of overall postoperative sequelae. The prevalence is slightly lower than data reported by Amarasooriya et al., which showed an overall complication rate of 25% [21]. We report 12% and 20.7% of complication rates related to DI and SI technique, respectively, with no statistically significant correlation to the adopted procedure. According to our data, SI technique may lead to a slightly higher number of postoperative complications in line with some literature data confirming the lower prevalence of sequelae after DI approach [19, 22, 23]. The LABCN was the most commonly injured nerve, with a rate between 5 to 57% [24]. In our cohort, LABCN neuropraxia is more frequent in the SI technique contrary to the series of Lang et al., which found a higher rate in DI technique [20]. Furthermore, some systematic reviews and meta-analysis of comparative studies described a higher rate of LABCN injury in SI technique, confirming our results [13, 21]. The LABCN is particularly vulnerable in the SI approach, during preparation of the bicipital tuberosity due to the nerve being retracted [21]. We do not report any case of tendon rerupture, differently from the majority of literature data which reported a complication rate ranging between 1.5 and 5.4% [21, 24, 25]. We register 1 case of radioulnar synostosis correlated to DI technique (4%). According to the literature, the synostosis complication was almost exclusively collected after DI approach [12, 26]. With this regard, Boyd and Anderson technique was associated with a rate of synostosis between 1 and 8% [12, 26]; then, the modification of the procedure provided by Morrey [4] reduced the rate of this complication, but did not eradicate it. In the present analysis, 4 patients (7.4%) have non-symptomatic HO. Similarly, some authors reported a comparable prevalence of HO ranging between 5 and 10% [26, 27]. Among the 4 patients presenting with HO, 3 performed SI technique. Contrary to our results, Amarasooriya et al. registered a similar incidence of HO for all fixation techniques, other than the cohorts of patients treated with interference screws or cortical buttons [21]. In the present study, the higher rate of HO in the SI approach is in disagreement with other authors [19, 28] who described a significant correlation between HO and the DI technique.

Overall, we report a lower rate of complications associated with the DI technique according to the findings of Kodde et al. [29]. The aforementioned systematic review highlighted that the double-incision procedure may be a safer approach than previously thought [29]. Similarly, Giacalone et al. [6] defined the DI technique as a relatively safe and non-invasive procedure as well as costless. The main limitations of this study are its retrospective nature associated with a relatively small sample of patients and the choice of surgical technique based on the preference of the surgeon. While the numbers are not ideal, they represent an important value if the short timeline is taken into consideration. Some positive aspects of this analysis are as follows: 2 standardized techniques, always performed by the same two surgeons, associated with homogeneous rehabilitation, which allows us to directly compare and validate results and application of international scores (DASH and MAYO) collected at the last FU, which permits a direct comparison with different reports in the literature.

## Conclusion

A consensus regarding the optimal surgical management of distal biceps tendon ruptures is still missing. According to our data, SI technique may lead to a higher number of postoperative complications. On the other hand, the DI approach can be considered as a safe and non-invasive technique associated with fewer sequelae and satisfying clinical results. To our knowledge, the SI and DI techniques provide a reliable and strong repair with an optimal recovery of ROM returning to previous activity with substantially overlapping timelines. The present study suggests that the treatment decision making should be driven by the preference of a highly experienced orthopedic surgeon taking into account the slightly different complication rate of the two techniques.

## Data Availability

All data are available in the main text and tables. Additional information can be provided if solicited.
