# Correction to: Acute distal biceps tendon rupture: retrospective analysis of two different approaches and fixation techniques

**DOI:** 10.1007/s00590-021-03152-4

**Published:** 2021-10-22

**Authors:** Marco Distefano, Lorenzo Sensi, Leonardo di Bella, Raffaele Tucci, Efisio Bazzucchi, Luigi Zanna

**Affiliations:** Department of Shoulder and Elbow, University of Florence, A.O.U. Careggi CTO - Largo Palagi 1, 50139 Florence, Italy

## Correction to: European Journal of Orthopaedic Surgery & Traumatology 10.1007/s00590-021-03132-8

The original version of this article unfortunately contained a mistake. Family name of the first author was incorrectly written as Di Stefano.

The correct name should be Marco Distefano.

